# Kinematic analysis of motor learning in upper limb body-powered bypass prosthesis training

**DOI:** 10.1371/journal.pone.0226563

**Published:** 2020-01-24

**Authors:** Conor Bloomer, Sophie Wang, Kimberly Kontson

**Affiliations:** 1 Division of Biomedical Physics, Food and Drug Administration, Silver Spring, Maryland, United States of America; 2 Department of Bioengineering, University of Maryland, College Park, Maryland, United States of America; Manchester Metropolitan University - Cheshire Campus, UNITED KINGDOM

## Abstract

Motor learning and compensatory movement are important aspects of prosthesis training yet relatively little quantitative evidence supports our current understanding of how motor control and compensation develop in the novel body-powered prosthesis user. The goal of this study is to assess these aspects of prosthesis training through functional, kinematic, and kinetic analyses using a within-subject paradigm compared across two training time points. The joints evaluated include the left and right shoulders, torso, and right elbow. Six abled-bodied subjects (age 27 ± 3) using a body-powered bypass prosthesis completed the Jebsen-Taylor Hand Function Test and the targeted Box and Blocks Test after five training sessions and again after ten sessions. Significant differences in movement parameters included reduced times to complete tasks, reduced normalized jerk for most joints and tasks, and more variable changes in efficiency and compensation parameters for individual tasks and joints measured as range of motion, maximum angle, and average moment. Normalized jerk, joint specific path length, range of motion, maximum angle, and average moment are presented for the first time in this unique training context and for this specific device type. These findings quantitatively describe numerous aspects of motor learning and control in able-bodied subjects that may be useful in guiding future rehabilitation and training of body-powered prosthesis users.

## Introduction

Upper limb amputees experience well-documented and self-reported disability, loss of function, and over-use injury with the loss of the upper extremity [1, 2]. With this loss, individuals are faced with adapting and relearning aspects of motor control with the reduced or less easily controlled degrees of freedom (DOFs) of a prosthetic device. While the functional and motor performance of other rehabilitation patient populations have been studied extensively (e.g. stroke [3–5]), the dynamic progress and motor learning of upper limb amputees has been relatively neglected. Meanwhile, an emergence of quantitative movement-based measures, enabled by motion capture technology, have gained popularity and have allowed for reliable study of movement and motor control. This study aims to address the gap in our understanding of the dynamics of motor learning and control in the upper limb body-powered (BP) prosthesis user by examining a series of kinematic and kinetic variables during prosthesis training. We believe our choice of measures and unique examination of novel users during device training will provide new insights into trainings effects and may guide future rehabilitation practices.

Integrating a prosthetic device into daily use requires adapting and relearning aspects of upper limb control, or more generally motor learning. Motor learning can be defined as the improvement of accuracy, quickness, smoothness, or efficiency for complicated movements [6]. In the upper limb specifically, Bernshtein’s”DOF problem” (i.e. many movement are available to an individual that result in the same action) is frequently cited and accepted to explain motor learning [7, 8]. This theory states that the role of the central nervous system in motor learning is to control redundancy by selecting and limiting the DOFs used to complete an action. This means, in part, the amputee is faced with relearning which available DOFs will provide the most accurate, quick, smooth, or efficient movement. For the prosthesis user, this process is complicated by the loss of distal DOFs and the incorporation of prosthetic DOFs.

While improved accuracy, quickness, smoothness, and efficiency are important variables in motor learning and rehabilitation generally, the unique DOF problem amputees face introduces the additional motor control concern of compensation. Compensatory movements encapsulate amputees’ tendencies to rely on proximal DOFs to regain function [9]. In the transradial and transhumeral amputee, compensation favors the use of trunk and shoulder movements as opposed to distal prosthesis movements. While compensatory movements can be considered adaptive and help return function, there is concern that they may be connected to the prevalence of overuse injuries and structural changes observed in the spines of upper limb amputees [1, 2]. Therefore, it is important to understand not only traditional motor learning in terms of accuracy, quickness, smoothness, and efficiency but also how compensatory movement and relative joint contributions in the upper limb prosthesis user change with prosthesis training.

Fortunately, motor learning and performance is well understood in the broader rehabilitation population. Accuracy, quickness, smoothness, and efficiency have been quantified using motion capture as a distance from a target location, movement velocity, normalized jerk, and path length respectively in numerous patient populations [3, 10–12]. In the stroke patient population for example, normalized jerk has been shown to decrease, indicating smoother movement, with training during a reaching task [13]. Efficiency has been similarly shown to increase with practice, as measured by the path length of the hand’s trajectory during task completion, in stroke patients [14]. Quickness is also well established in rehabilitation research and amputee performance assessment. Novel prosthesis users were able to perform tasks in less time with additional training in several studies [6, 15, 16]. Given these results, we expect prosthesis users would demonstrate improved quickness, smoothness, and efficiency as motor learning occurs with training. However, to our knowledge no study has yet demonstrated these changes together in a novel BP prosthesis user specifically.

More specific aspects of motor control, such as compensatory movement, are less generalizable and therefore more difficult to predict. A number of studies have established range of motion (ROM) and maximum angle as joint specific measures of compensation [9, 17, 18]. In a study of myoelectric and BP prosthesis users, increased ROM was shown in the shoulder and trunk, suggesting compensation at these joints, compared to able-bodied controls [9]. Similarly, reducing distal DOFs through bracing has also been shown to alter shoulder ROM and increase maximum angle [17]. Measuring the strain or work of individual joints may also be useful as a measure of a joint’s relative contribution to movement or risk of overuse injury [19, 20]. Drawn from sports medicine and rehabilitation, these studies have established joint moments during movement as a potential indicator of injury risk and have distinguished results based on the movement strategy employed. However, these studies have not explicitly explored how these variables change with training. Work by Thies et. al has provided some insight by demonstrating decreased joint angle variability during task performance with training of myoelectric users, suggesting users settle on a common strategy with increased practice [21]. Qualitative work by Resnik has also demonstrated improved movement quality and reduced compensation with training in DEKA Arm users [22]. However, these trends have not been demonstrated yet in the BP user independently during training. We expect that compensatory movements will initially decrease (reflected by decreased ROM, maximum angle, and moment at the shoulder and trunk) as users train and become more proficient with the distal DOFs of the prosthesis.

Considering trainings proven impact on functional performance and device acceptance, as well as the current state of upper-limb amputee outcomes, we are presenting a kinematic study of the effect of prosthesis training on several motor learning variables [23–30]. Specifically, we asked whether changes occurred in traditional motor learning variables, such as quickness, smoothness, and efficiency, and in compensatory movement variables, such as range of motion, maximum angle, and joint moment, following five and ten training sessions using a within-subject paradigm. We hypothesized time required to complete a task would decrease as well as normalized jerk and path length for each joint and task. We also hypothesized that ROM, maximum angle, and joint moment would decrease for the shoulder and trunk as users reduce their dependence on compensatory movements for each task. Our findings aim to highlight the importance of understanding the motor control and learning implications of training and the utility of quantitative measures in the field.

## Methods

### Participants

**Fig 1:**
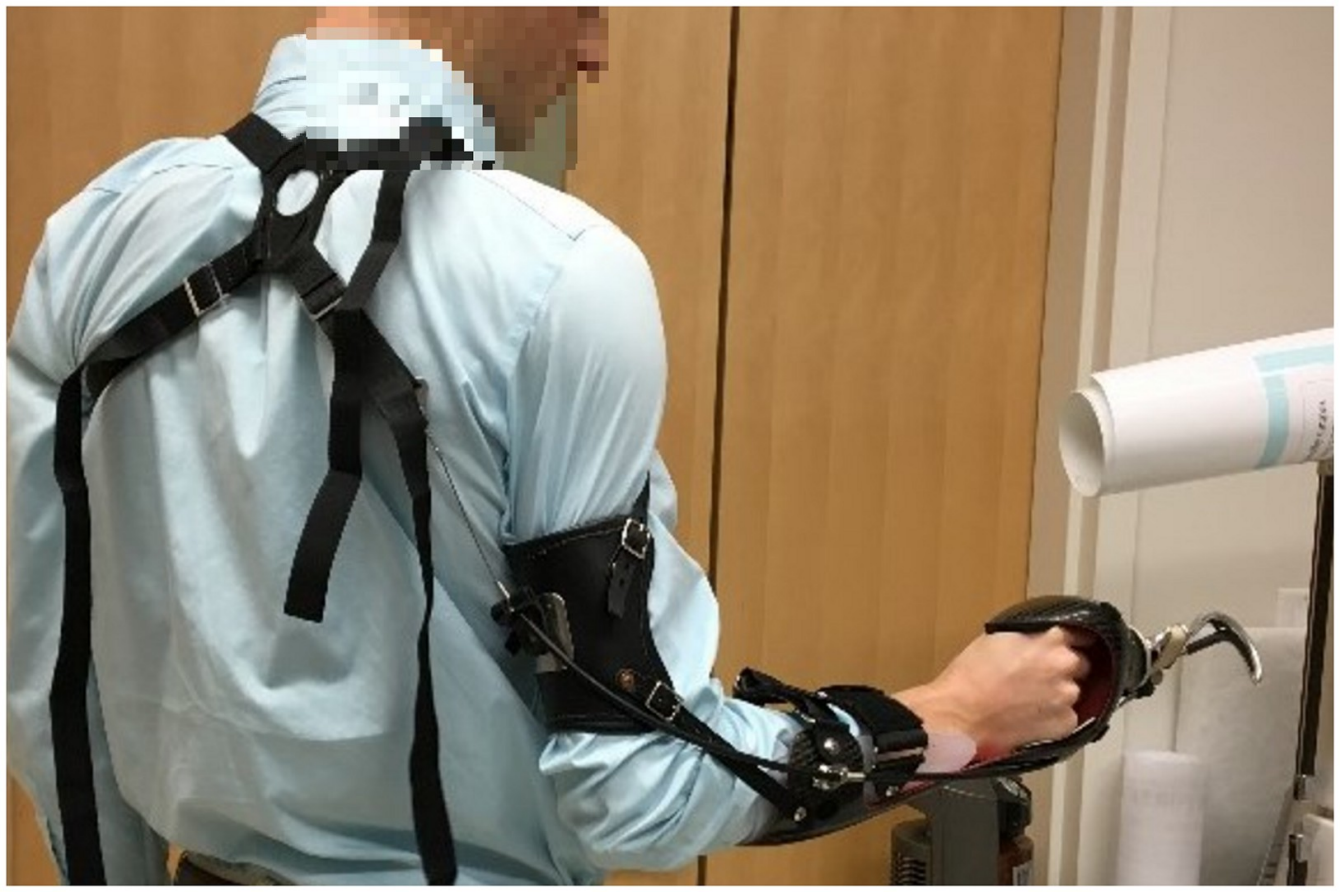
Bypass prostheses. Donned body-powered bypass prosthesis with harness shown.

Six right-handed subjects (three female, three male; mean age ± standard deviation; 28.67 ± 3.27 years) with no upper limb disability or impairment participated in the study. Each participant trained on a bypass prosthesis to facilitate the inclusion of novel users. A bypass prosthesis is an equivalent prosthetic device that allows a non-disabled user to activate a terminal device with similar controls that an amputee would use to operate a custom-made prosthesis. Arm Dynamics (Dallas, TX) provided the BP bypass prosthesis, featuring a voluntary opening Hosmer 5X hook terminal device, with manual wrist rotation, and a figure-of-eight harness (Fig 1A). The bypass prosthesis was designed with a distal offset of 12 cm.

We recognize our sample was limited by the extensive time commitment required and have included multiple trials, tasks, and segments, described later, into our design to provide a more robust repeated measures analysis. Subjects were not compensated for their participation. This study was approved by the Institutional Review Board of the U.S. Food and Drug Administration (Protocol Number #14-086R). All subjects provided written informed consent prior to participating in the study.

### Training

Training was administered in 10 two-hour sessions according to the referenced protocol outlined in greater detail by Bloomer, et. al [15]. Initial content included a device orientation and use checkpoint during which participants were taught how to operate the bypass prosthesis (orientation) and were consequently asked to demonstrate operation (checkpoint). The bulk of training consisted of guided practice in object manipulation tasks and activities of daily living (ADLs) as well as unstructured, self-guided practice, referred to as free training. Each of these training activities, guided and free training, were allotted in each session. The tasks and activities used during training and their presentation across participants were strictly controlled, including administrator feedback limited to reminders to use prepositioning. Readers are encouraged to review the referenced protocol for greater detail.

### Data collection and functional tests

Following training sessions five and ten, motion capture data were collected from each participant as they performed a different set of tasks from those performed in the training sessions, resulting in two separate motion capture sessions. The two tests used to assess function and provide data for kinematic analysis were the Jebsen-Taylor Hand Function Test (JHFT) and the targeted Box and Blocks Test (tBBT) [31, 32]. Analyses were limited to just three of the seven JHFT tasks: JHFT task 2 –simulated page turning, JHFT task 4 –simulated feeding, and JHFT task 7 –lifting large heavy objects. JHFT task 1 –writing and JHFT task 3 –picking up small objects were excluded due to previously demonstrated high intra-subject kinematic variability, while JHFT task 5 –stacking checkers and JHFT task 6 –lifting large light objects were excluded to avoid redundancy in task demands [18]. The tBBT was chosen as a more ecologically valid variation of the Box and Blocks Test commonly used in motion capture studies [31]. The test is an abstract object transport task which features 16 blocks on one side of a partition arranged in a 4x4 array. Participants are required to pick up, transport, and control the placement of these blocks on the opposite side of the partition. All JHFT tasks were performed in a seated position. The tBBT was performed in a standing position. For those seated tasks, subjects were asked to start with both hands resting on the height-adjustable table, positioned such that the elbow made a 90° angle when the subjects’ hands were resting palm down on the surface of the table. For the tBBT task, the height of the table was adjusted to be 10 cm below the subjects’ anterior superior iliac crest when the subject was standing. For all tasks, subjects performed two trials and were instructed to complete the task as quickly and as accurately as possible.

The data from the motion capture sessions are the source of data presented in this paper and are referred to as session one (S1) and session two (S2) throughout the rest of the paper. Movements were captured using an optical motion analysis system from Vicon (Vicon, Oxford, UK) consisting of eight B10 Bonita cameras (Fs = 100 Hz). Camera positions were optimized to a 1.2 x 1.2 x 2.4 meter capture volume and calibrated per the manufacturer’s specifications. Frontal recordings of subjects were also collected using a digital video camera. For analysis, the Plug-in-Gait upper body model from Vicon was used [33]. In all, 27 reflective markers were placed on the subject per model documentation and subject specific measurements were recorded for model calibration.

### Kinematic analysis

For our analysis, the following joints and DOFs were analyzed: left and right shoulder flexion/extension, left and right shoulder abduction/adduction, left and right shoulder rotation, right elbow flexion, torso forward flexion, torso lateral flexion, torso rotation. Right shoulder and torso DOFs are implicated in compensatory movements, while the contralateral left shoulder is of interest given the activation method of the BP device [9]. Joint angles were calculated using YXZ Euler angle decomposition for each task and trial. Torso angles were computed relative to defined planes in the global coordinate system. Elbow flexion and shoulder angles were computed relative to defined body segments. Calculations of kinematic parameters have been previously described in greater detail [34–36].

Data were filtered with a 4^th^ order, zero lag, lowpass Butterworth filter at 6 Hz. Kinetic modeling required calculations of mass and radii of gyration. Center of mass for each body segment was defined by a proportion along the segment distal from the joint center. The mass of each body segment was calculated in Vicon as a proportion of inputted body mass, taken at the time data collection, for all analyses.

Tasks were divided into several repetitive segments, where segment start was marked by the initiation of the approach to manipulate the object in question, and segment end was defined as the release of that object. Task analyses therefore include multiple segments treated as equivalent trials. All segments were time normalized to a common number of samples between sessions to facilitate analyses and comparisons.

### Metrics

To evaluate motor learning and compensatory movement over the course of training with the BP bypass prosthesis, several metrics were used. Metrics are drawn from features of the collected joint angle versus time data sets for each task and joint. Due to the high number of joints, tasks, and analyses, joints with 3 DOFs were reduced into a single resultant angular trajectory versus time for all measures. Resultant trajectories (T) were calculated as the square root of the square of vector sum of the angular value (θ_x_) for each DOF at a particular time point to generate a single vector, where x represents a DOF (Eq 1). This single vector essentially described the radial distance of the joint from the neutral position where all angles equal zero.

$$T(i)=\sqrt{{{\theta (i)}_{1}}^{2}+{{\theta (i)}_{2}}^{2}+{{\theta (i)}_{3}}^{2}}$$ (1)

We consider quickness, smoothness, and efficiency to be the major parameters in understanding motor learning in this study. Quickness was measured by time to completion of the various tasks in the previously described functional tests: JHFT and tBBT. Smoothness was measured by normalized jerk [37–40]. Jerk is defined as the derivative of acceleration. Normalized jerk (NJ) is calculated by finding the time integral of squared jerk and dividing by *length*^*2*^*/duration*^*5*^ of the movement to remove the influence of both distance and time [40, 41]. The equation applied to normalized jerk can be found in Eq 2, where N is the length of the *T* vector.

$$NJ=\sqrt{\frac{1}{2}\sum_{i=1}^{N}{T(i)'''}^{2}*\frac{{duration}^{5}}{{length}^{2}}}$$ (2)

To assess efficiency, path length (PL) between S2 and S1 was compared [9, 11, 12]. Here, path length refers to the distance tracked along the three-dimensional joint angular trajectory for an individual segment (Eq 3).

$$PL=\int_{i=1}^{N}\left|T\;(i+1)-T\;(i)\right|di$$ (3)

To assess motor control in terms of compensatory movements, we considered several kinematic and kinetic measures. ROM of our resultant angular trajectory data was used to summarize the subject’s working space. The increase or decrease of ROM in the right shoulder and torso across sessions is considered indicative of increased or decreased compensatory movement. We also extracted the maximum angle from each resultant angular trajectory indicating the extremes of joint use and potential compensation, interpreted similarly to ROM[9, 42]. The strain at the joint is quantified by the average moment. This analysis examines individual joint use but uniquely address potential indicators of overuse and musculoskeletal strain. Average moment was calculated from outputted joint kinetics and is reported here as an average of the resultant joint moment trajectory across a segment. This value characterizes the physical stress placed on the joint during a task, which when compared across sessions can demonstrate increased or decreased joint effort [19, 43–45].

In addition, a series of novel or peripheral measures are included as supplemental materials with associated methods and results. These measures include functional volume, a Pearson’s correlation coefficient analysis, and path integral. We believe these measures provide interesting and novel insights into upper-limb motor learning and control but given their novelty in the literature, they are included more appropriately as supplemental material.

### Statistical analysis

A within-subject paradigm was used to assess differences across training for each metric. Results are calculated as the difference between S2 and S1. Difference calculations are further matched within-subject for task, trial, and segment. To summarize trends within task, joint, and measure, results are grouped accordingly and reported as medians and interquartile ranges. A Wilcoxon signed rank test for zero median was used to assess significance of any increases or decreases in metrics between sessions. This non-parametric test was used due to potential violation of the normality assumption required for parametric statistical tests and a small sample size. This two-sided signed rank test examines whether a single sample, difference of the paired S2 and S1 data sets, comes from a zero-median distribution, representing no change between sessions, and returns the p-value. P-values below 0.05 were considered significant. Due to the small sample size (12 trials across 6 subjects) p-values below 0.01 and 0.001 were not further differentiated.

To effectively show the impact of training between sessions, the results are presented as boxplots of the difference distribution between S2 and S1 of each metric. Metric values from S1 were always subtracted from S2, meaning a median value > 0 indicates the metric was higher in S2 compared to S1. Conversely, a median value < 0 indicates the metric was lower in S2 compared to S1. To facilitate the visualization of these differences, boxplots were colored blue if the median < 0, and red if the median > 0. The line y = 0 is also shown on each plot. Stars indicate significant separation from a zero median, positive or negative, with p values less than 0.05 indicated with a single star. The X axis indicates tasks abbreviated to JHFT 2, JHFT 4, JHFT 7, and tBBT for JHFT task 2 –simulated page turning, JHFT task 4 –simulated feeding, JHFT task 7 –lifting large heavy objects, and targeted Box and Blocks Test, respectively.

## Results

### Motor learning

**Fig 2:**
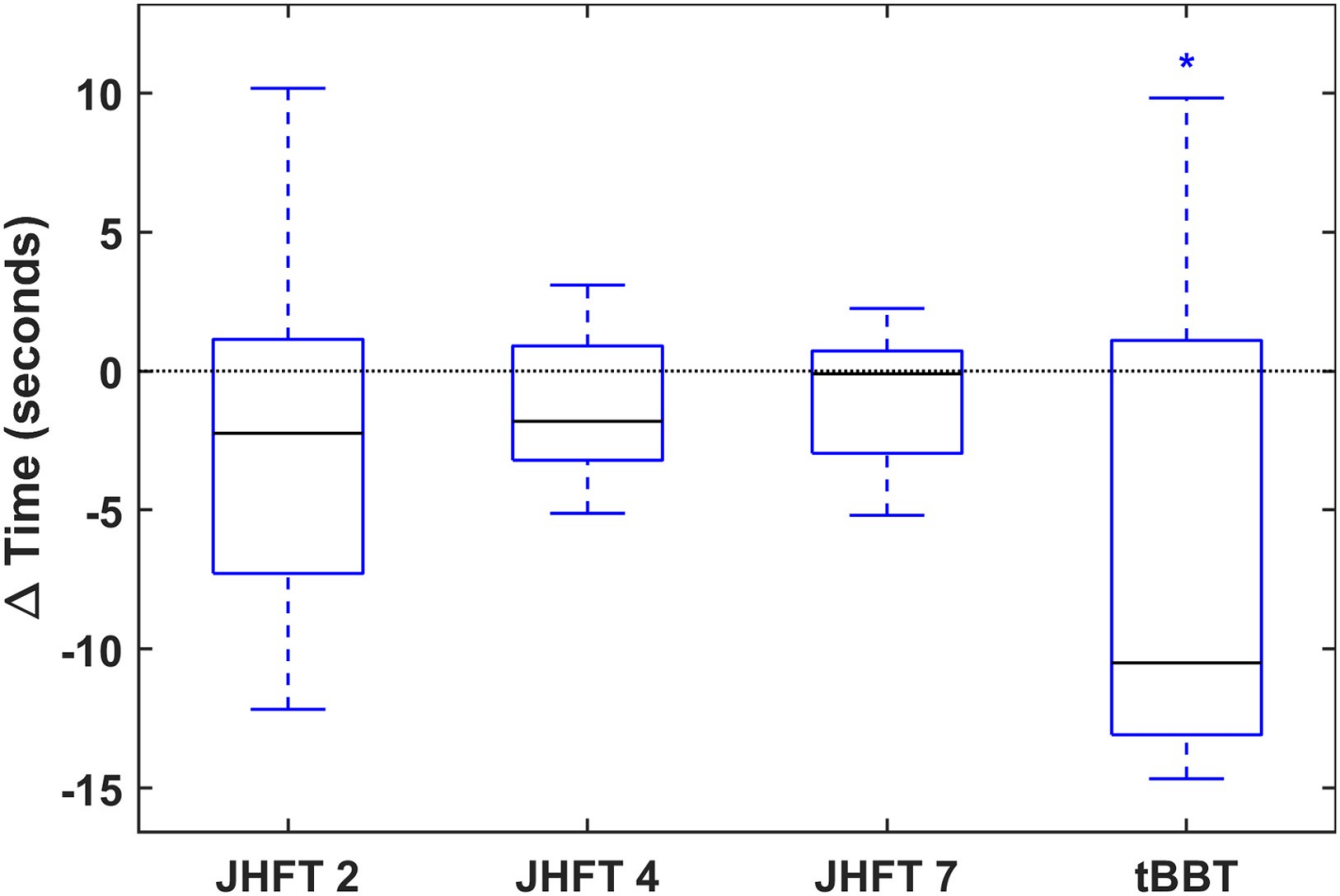
Quickness (time to completion). Differences in task completion times between sessions in median and quartile box plot. Blue boxes indicate medians below zero, or improved quickness, red boxes indicate medians above zero, or decreased quickness. X axis ticks indicate JHFT task number (2, 4, and 7) and tBBT.

To assess motor learning, we first report changes in quickness across sessions. Results indicate that BP bypass prosthesis users completed the tasks faster with additional training. The distribution of these calculated differences for each task are displayed as a box plot (Fig 2). A significant decrease in time to completion was found for the tBBT in particular. The remaining three tasks trended similarly, demonstrating improved quickness as well. Across all tasks a median reduction of 1.87 seconds and an interquartile range of 6.85 seconds was observed.

**Fig 3:**
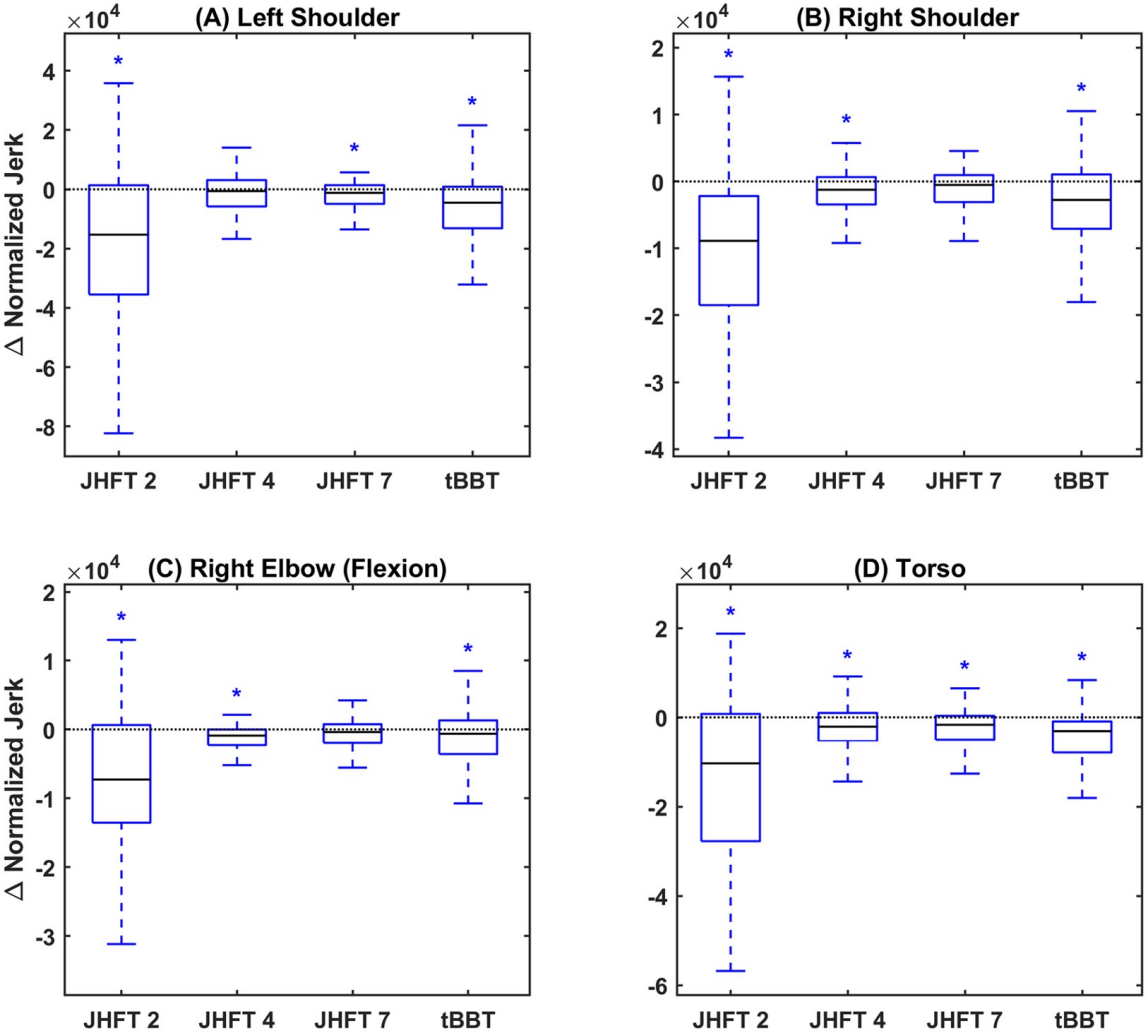
Smoothness (normalized jerk). Difference in normalized jerk between sessions summarized as a boxplot for (A) left shoulder joint, (B) right shoulder joint, (C) right elbow joint, and (D) torso. Blue boxes indicate medians below zero, or improved smoothness, red boxes indicate medians above zero, or decreased smoothness. X axis ticks indicate JHFT task number (2, 4, and 7) and tBBT.

The difference distributions for normalized jerk for each joint and task are shown in (Fig 3). Results show decreasing normalized jerk values from session 1 to session 2 for all tasks and joints, demonstrating improved smoothness. Improvements in smoothness were significant in all but three instances: left shoulder joint for JHFT task 4 –simulated feeding, and right shoulder joint and right elbow flexion for JHFT task 7 –lifting large heavy objects.

**Fig 4:**
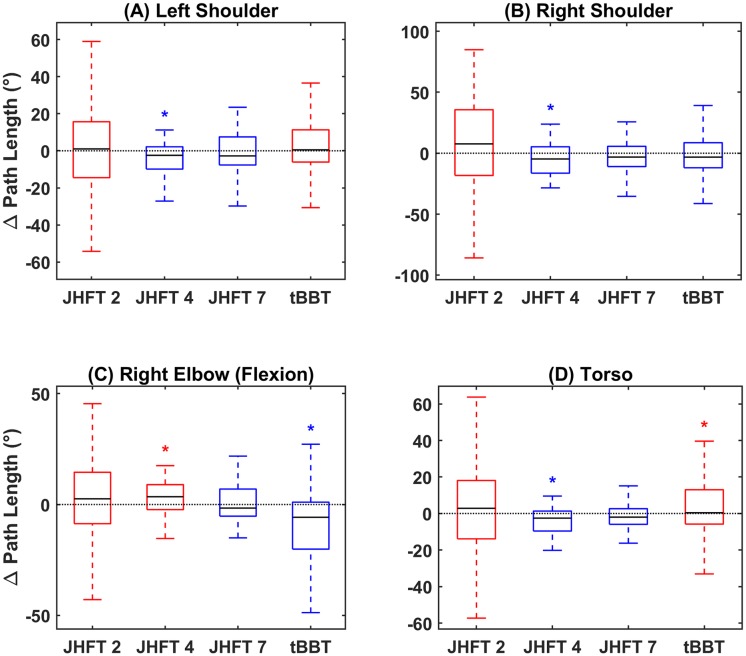
Efficiency (path length). Difference in path length between sessions summarized as a boxplot for (A) left shoulder joint, (B) right shoulder joint, (C) right elbow joint, and (D) torso. Blue boxes indicate medians below zero, or improved efficiency, red boxes indicate medians above zero, or decreased efficiency. X axis ticks indicate JHFT task number (2, 4, and 7) and tBBT.

Path length results representing efficiency were more variable than the preceding two motor learning characteristics and their metrics. This difference is evident for all joints (Fig 4), which demonstrate both positive and negative medians in every joint subplot. For three out of four tasks, the right shoulder joint showed a decrease in pathlength with training (Fig 4B). At both the left shoulder joint and torso, significant decreases in pathlength were seen during performance of the JHFT4 –simulated feeding task. For most other tasks at the left shoulder and torso (except tBBT), there were no significant differences in pathlength with training. Pathlength of the right elbow join significantly increased with training for the JHFT 4 –simulated feeding tasks, and significantly decreased with training for the tBBT.

### Compensatory movement

**Fig 5:**
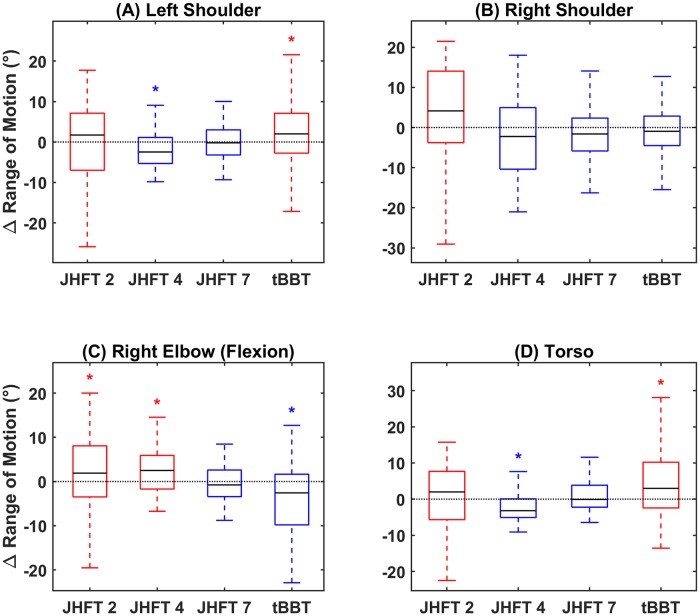
Range of motion difference in range of motion between sessions summarized as a boxplot for (A) left shoulder joint, (B) right shoulder joint, (C) right elbow joint, and (D) torso. Blue boxes indicate medians below zero, or decreasing ROM, red boxes indicate medians above zero, or increasing ROM. X axis ticks indicate JHFT task number (2, 4, and 7) and tBBT.

For compensatory movement, several measures were used. We first report on ROM as a summary of joint movement. At the right shoulder joint, all tasks except JHFT 2 –Page Turning showed a decrease in ROM with training (Fig 5B). However, none of those results were significant. At both the left shoulder joint and torso, a significant decrease in ROM was seen during performance of JHFT 4 –Simulated Feeding, indicating a decrease in compensatory movement strategy with training for this task (Fig 5A and 5D). Conversely, at these same joints, a significant increase in ROM was seen across all subjects performing the tBBT (Fig 5A and 5D) At the right elbow joint, a significant increase in ROM with training was seen for JHFT 2 –Page Turning and JHFT 4 –Simulated Feeding while a significant decrease was seen with the tBBT (Fig 5C). Overall, the ROM metric was variable between tasks and joints.

**Fig 6:**
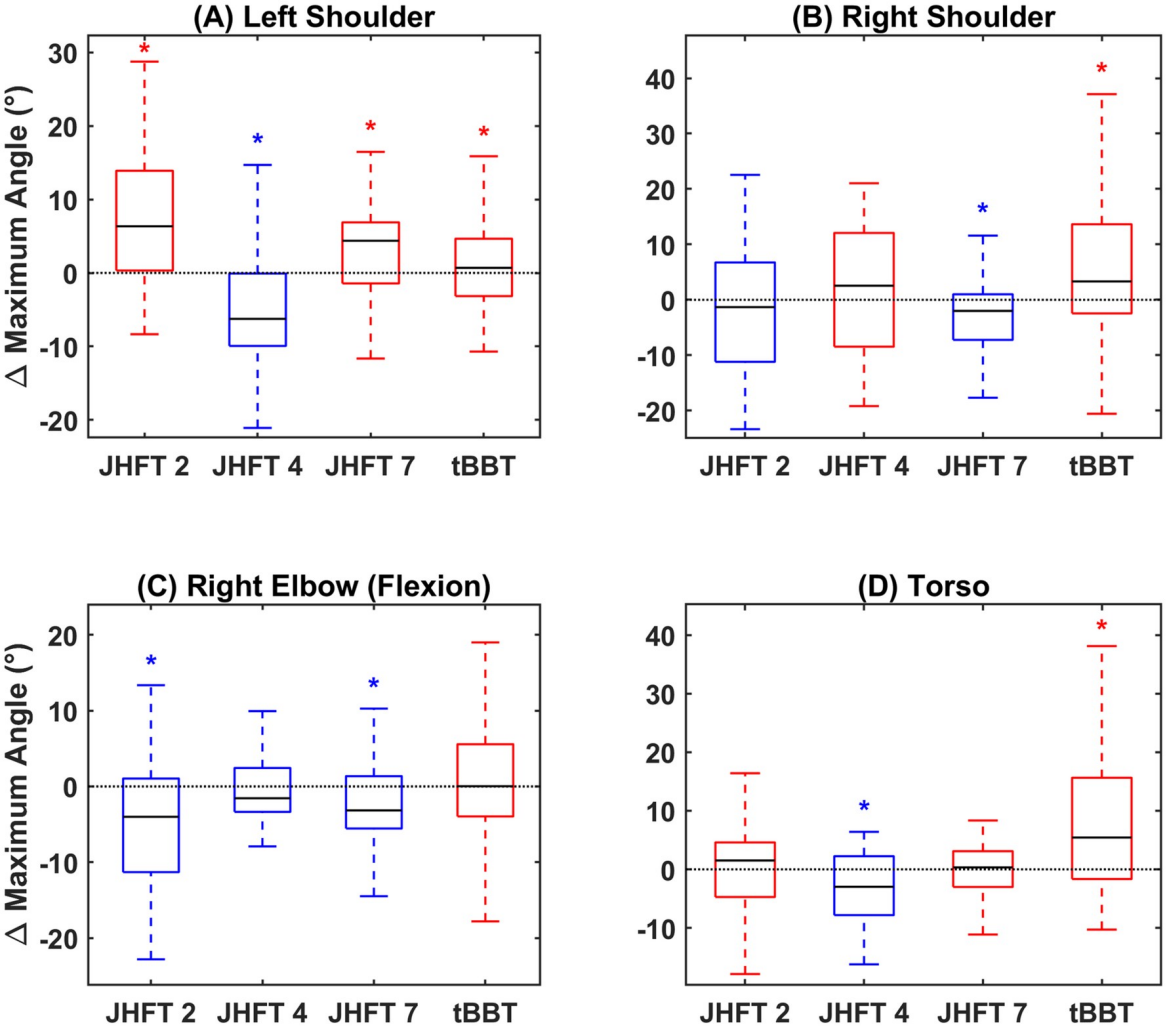
Maximum angle. Difference in maximum angle between sessions summarized as a boxplot for (A) left shoulder joint, (B) right shoulder joint, (C) right elbow joint, and (D) torso. Blue boxes indicate medians below zero, or decreasing maximum angles, red boxes indicate medians above zero, or increasing maximum angles. X axis ticks indicate JHFT task number (2, 4, and 7) and tBBT.

Distributions of maximum angle differences across sessions are shown in Fig 6. Differences during training in maximum angles were also variable across tasks and joints. At the left shoulder joint, significant increases in the maximum angle across training sessions during performance of all tasks except JHFT 4 –Simulated Feeding were observed (Fig 6A). At the right shoulder joint, results were split on the number of tasks showing an increase and decrease in ROM with training (Fig 6B). A significant increase in ROM with training was seen at the right shoulder joint for the tBBT and a significant decrease with training was seen with JHFT 7 –Lifting Heavy Cans. At the right elbow joint, a significant decrease in ROM was seen for JHFT 2 –Page Turning and JHFT 7 –Lifting Heavy Cans (Fig 6C). For most tasks, the torso also showed increases in ROM with training, with a significant increase during the tBBT (Fig 6D).

**Fig 7:**
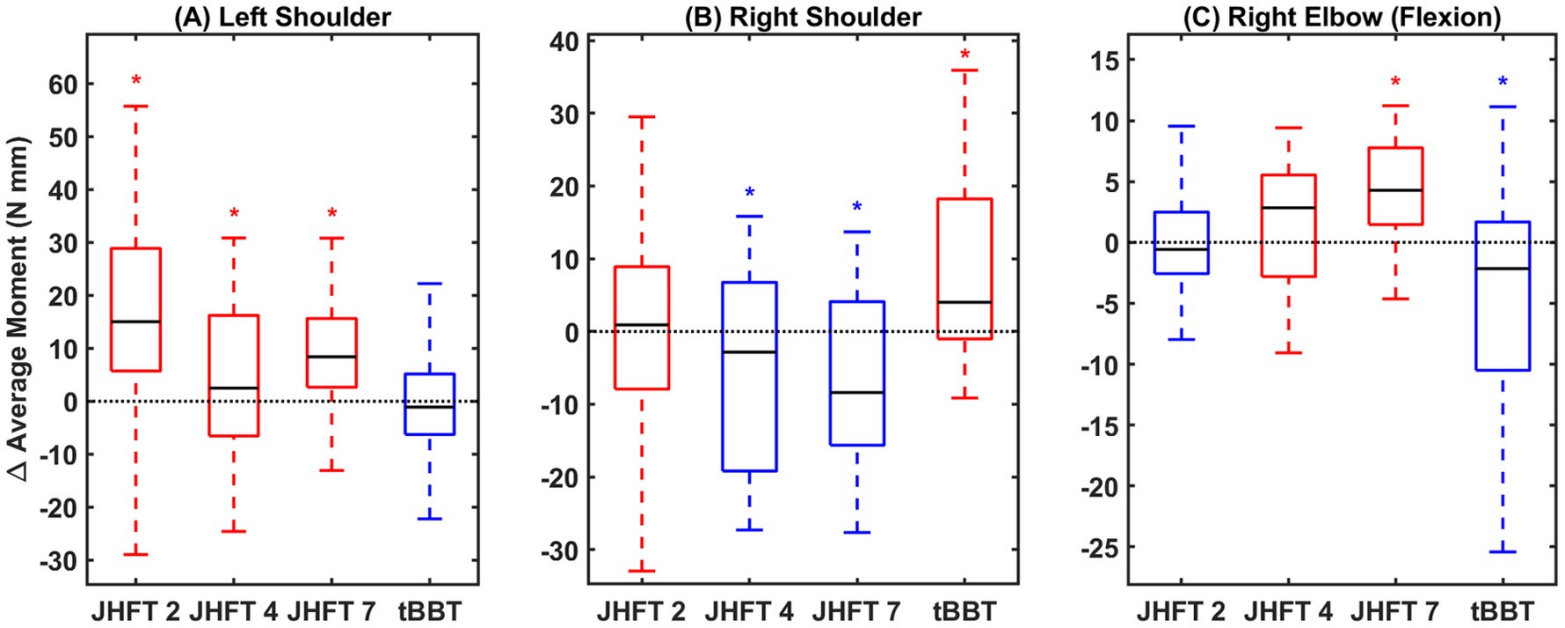
Average moment. Difference in average moment between sessions summarized as a boxplot for (A) left shoulder joint, (B) right shoulder joint, and (C) right elbow joint. Blue boxes indicate medians below zero, or reduced effort, red boxes indicate medians above zero, or increased effort. X axis ticks indicate JHFT task number (2, 4, and 7) and tBBT.

Finally, average moment is similarly reported to assess joint effort and compensation (Fig 7). Moment analysis was limited to those joints output by Vicon, which excludes the torso. For all tasks and joints, results were again highly variable outside of some limited trends. Exceptions include the left shoulder joint, which demonstrated increased moments for all tasks, except tBBT—stand. Notably, right shoulder angles and right elbow flexion appear to be inversely related.

## Discussion

This study demonstrates several means of characterizing movement in the upper limb prosthesis user and examines how these measures may collectively define motor learning in this particular population. Joint level analyses demonstrated training impacted the speed, smoothness, and efficiency of prosthesis users’ movements during task performance. Measures of compensatory movement also appeared to be affected by training with considerably more variability in joints and across tasks. These results shed light on the numerous effects of training and motor learning on the novel prosthesis user and highlight a motor control perspective that might inform future rehabilitation practice.

Results indicated traditional motor learning occurred in most measures in a variety of joints and across tasks (i.e. quickness, smoothness, and efficiency). While improvements in motor learning were generally expected, a joint level analysis had not been previously performed nor normalized jerk or path length metrics applied to this population and device type as measures of efficiency during training. This distinction revealed variability in efficiency that was not seen in quickness or smoothness results, challenging our hypothesis that traditional motor learning took place in our training context. Compensatory movement measures showed similar variability. While these measures were variable between tasks and joints, they still reflected significant changes across training suggesting they are sensitive to some aspect of training if not traditional motor learning, and may be useful in providing an objective and quantitative measure of motor learning for amputee rehabilitation.

The use of a joint level analysis specifically distinguishes distal and proximal segments of the limb which may be more important for the amputee population than the rehabilitation populations to which these measures were originally applied. Additionally, differences between joints, tasks, and motor learning measures highlight the complexity of motor learning. These differences suggest smoothness, quickness, and efficiency are not all measuring the same motor learning progression but may measure distinct aspects of it that can be treated distinctly in rehabilitation.

Efficiency, as measured by differences in joint path length, showed improvement with training but not to the extent of quickness or smoothness. Variability between tasks and joints was higher, suggesting efficiency may be a more complex and context specific aspect of motor learning. This measure may have been uniquely influenced by the kinetic chain of the upper limb. Movement in one joint may influence movement at another joint while completing a task. By isolating each joint in this chain, instead of analyzing a single distal trajectory as is common, our results may be difficult to interpret. Ultimately, an angular analysis of efficiency may not be suited for a study of this size.

Compensatory movement results were even more variable for tasks and in joints. While right shoulder and torso ROM trended downward across training collectively, the results were not overwhelming. Maximum angle and average moment results were similarly variable across joints and tasks. However, all measures did show significant changes in several observations suggesting they are capturing some dynamic of the prosthesis user. Considering the complexity and variety of the tasks analyzed, it may be that joint movements depend more on the demands of the tasks than the level of training achieved. Average moment results were particularly interesting despite not including the torso. Previous work has suggested prosthesis users use their shoulder while limiting their elbow movements compared to able-bodied users [9]. A similar inverse relationship between the elbow and shoulder is shown in our results. Additionally, these compensatory movement and motor learning results highlight not only variability between tasks, joints, and measures but within these dimensions. This fact is important to consider in the design of similar or future studies and may explain our difficulty in establishing trends within our data set.

The high variability seen in efficiency and compensatory movement results may be due to more conscious improvements or experimentation with movement strategy across training. During training, the research team commonly observed participants changing their approach to a task and their strategy in completing it. The modified approach and strategy may be due to the complexities and novelty of using a bypass prosthesis device and represent the “trial and error” period for the bypass prosthesis subjects to determine the optimal strategy and may represent similar “trial and error” periods for novel prosthesis users. These more gross motor changes are more likely reflected in gross motor measures such as range of motion and maximum angle as well as in joint path length and average moment. Alternatively, more fine motor improvements such as smoothness may occur as participants naturally grow more comfortable with the device regardless of movement strategy. Quickness may reflect a similar process, and because a joint level analysis was not performed the measure is not open to the same variability. In either measure, the results are less susceptible to changes in movement strategy whereas compensatory movement is defined by it. Therefore, for upper limb amputee rehabilitation and prosthesis training, it may be important to treat movement strategy and motor learning as separate processes. This idea is supported by anecdotal evidence of compensation where experienced users are observed with improved motor control while relying on poor or harmful movement strategies.

Unanimous motor learning results and unclear compensatory movement and efficiency results may also be the result of study limitations and prosthesis considerations. For the novel user a 10-session training regimen may simply be too short to capture the expected learning. While popularly cited, previous work suggesting this length was adequate did not feature the same objective measures used here. Additionally, our use of a bypass prosthesis is likely to have affected the movements we characterized in this study compared to those of an amputee user. It is possible that this difference may distinguish the motor learning observed from those of the intended amputee population.

While this study demonstrates important changes in motor control in novel prosthesis users it is ultimately limited by its smaller sample. Future work is needed to increase this sample and elucidate trends from variability with a more focused set of tasks and joint analyses. This could be facilitated by more targeted training sessions which could reduce the longitudinal commitment of subjects. The presentation of motor learning and control metrics in insolation also limits the interpretation of the work presented. A more in-depth subject-level analysis of the correlation between each motor learning and motor control metric could provide additional insight into the processes of learning. Additionally, future work is needed to establish and validate these rehabilitation metrics in the amputee population specifically.

Even as upper limb prostheses continue to advance, devices and outcomes continue to be unsatisfactory [29]. Training has a proven impact on device function and acceptance and is already widely accepted as part of prosthesis prescription [23–30]. By understanding motor learning with the commonly used BP prosthesis and what training’s effect is on performance, providers may be able to tailor rehabilitation more appropriately. This study demonstrates training’s impact on motor learning and compensatory movement and establishes a framework to improve our understanding further.

## Supporting information

S1 Fig Ellipsoid fit for a single subject’s left shoulder angular trajectory for tBBT. X, Y, and Z axes represent flexion/extension, abduction/adduction, and internal/external rotation, respectively.
(TIF)

S2 Fig Pearson’s correlation coefficient. Linear fit comparisons between sessions to generate R values summarized as a boxplot. The dotted line in each plot at y = 0.4 represents the R-value threshold above which waveforms are considered moderately to strongly correlated. Blue boxes indicate medians below threshold, or dissimilar strategies, red boxes indicate medians above threshold, or similar strategies. X axis ticks indicate JHFT task number (2, 4, and 7) followed by tBBT.
(TIF)

S3 Fig Path integral. Difference in path integral between sessions summarized as a boxplot. Blue boxes indicate medians below zero, or reduced effort, red boxes indicate medians above zero, or increased effort. X axis ticks indicate JHFT task number (2, 4, and 7) followed by tBBT.
(TIF)

S1 Table Median difference of ellipsoid centroid location and volume for each joint and task. Significance between motion capture Session 1 and Session 2 is indicated by a * signifying a p-value < 0.05.
(DOCX)

S1 File (DOCX)
