# Supplementary figures and images for: Kinematic analysis of motor learning in upper limb body-powered bypass prosthesis training
Source: PLoS One. 2020 Jan 24;15(1):e0226563. doi: 10.1371/journal.pone.0226563 (PMC6980621; doi:10.1371/journal.pone.0226563)

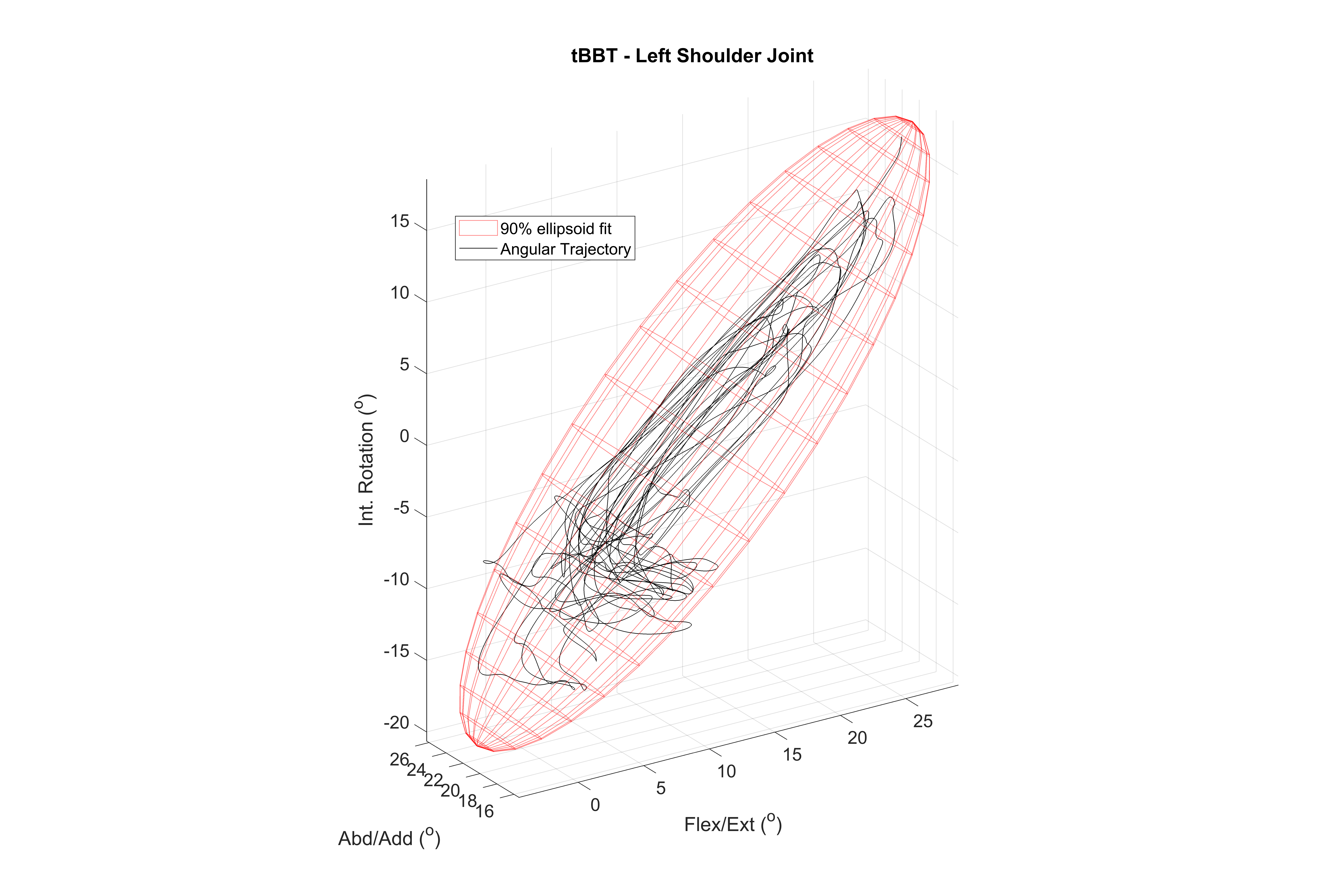

Supplement: S1 Fig — X, Y, and Z axes represent flexion/extension, abduction/adduction, and internal/external rotation, respectively. (TIF) [file pone.0226563.s001.tif]

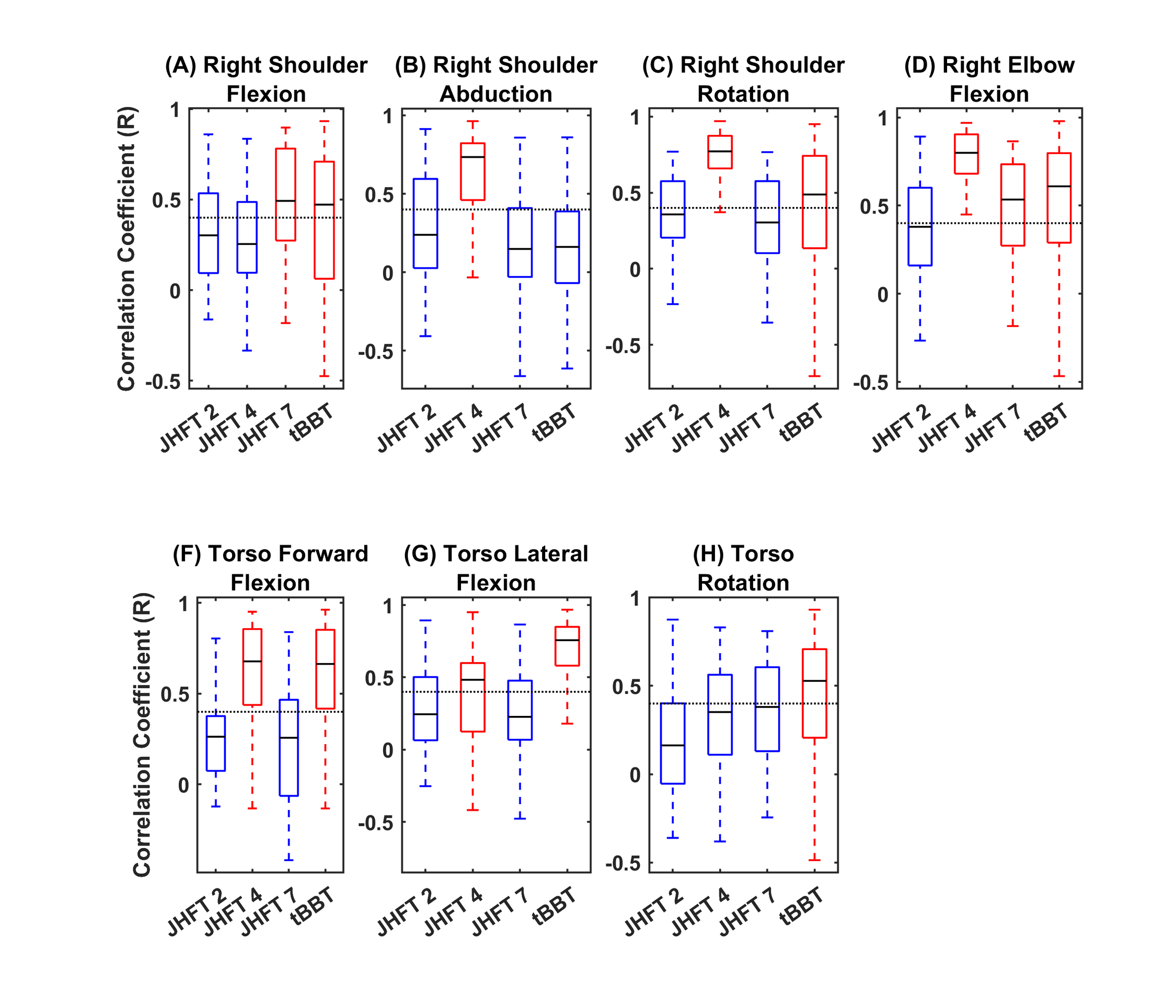

Supplement: S2 Fig — Linear fit comparisons between sessions to generate R values summarized as a boxplot. The dotted line in each plot at y = 0.4 represents the R-value threshold above which waveforms are considered moderately to strongly correlated. Blue boxes indicate medians below threshold, or dissimilar strategies, red boxes indicate medians above threshold, or similar strategies. X axis ticks indicate JHFT task number (2, 4, and 7) followed by tBBT. (TIF) [file pone.0226563.s002.tif]

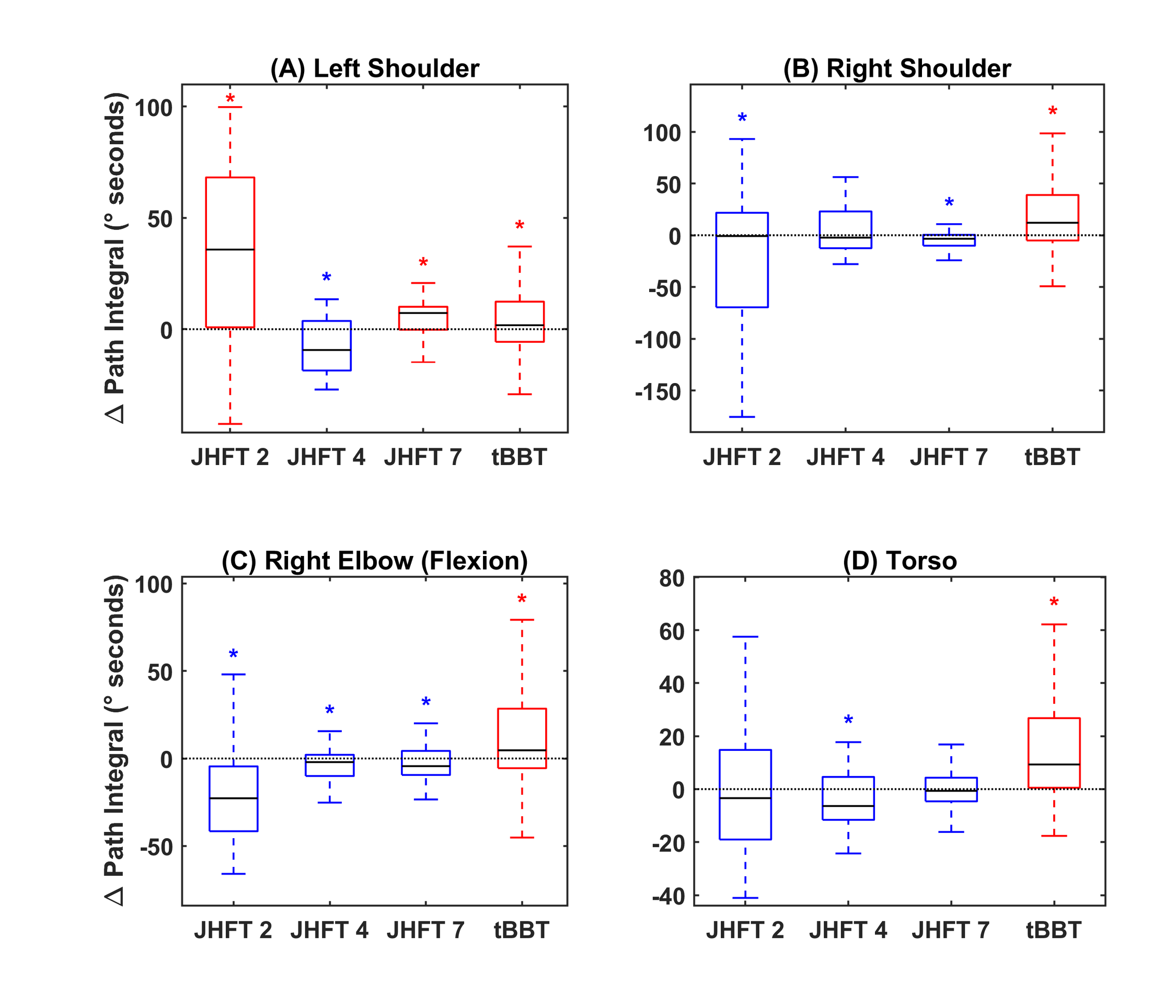

Supplement: S3 Fig — Difference in path integral between sessions summarized as a boxplot. Blue boxes indicate medians below zero, or reduced effort, red boxes indicate medians above zero, or increased effort. X axis ticks indicate JHFT task number (2, 4, and 7) followed by tBBT. (TIF) [file pone.0226563.s003.tif]
