# Supplementary material for: Kinematic analysis of motor learning in upper limb body-powered bypass prosthesis training
Source: PLoS One. 2020 Jan 24;15(1):e0226563. doi: 10.1371/journal.pone.0226563 (PMC6980621; doi:10.1371/journal.pone.0226563)
Supplement: S1 Table — Significance between motion capture Session 1 and Session 2 is indicated by a * signifying a p-value < 0.05. (DOCX) [file pone.0226563.s004.docx]

**Supplemental Table 1:** Median difference of ellipsoid centroid location and volume for each joint and task. Significance between motion capture Session 1 and Session 2 is indicated by a * signifying a p-value < 0.05.

|  |  | **Difference in centroid** | | | | | | **Difference in volume** | |
| --- | --- | --- | --- | --- | --- | --- | --- | --- | --- |
|  |  | *x med* | *x IQR* | *y med* | *y IQR* | *z med* | *z IQR* | *med* | *IQR* |
| **Left Shoulder** | *tBBT* | 8.3 * | 9.5 | 2.6 * | 3.8 | -7.0 | 8.8 | -672.7 | 10373.3 |
|  | *JHFT task 2* | 12.6 | 13.4 | 4.0 | 9.4 | 4.8 | 30.1 | 4209.3 | 9929.7 |
|  | *JHFT task 4* | 3.5 | 10.1 | -2.5 | 11.5 | 5.5 | 25.8 | -53.7 | 750.5 |
|  | *JHFT task 7* | 3.1 * | 6.3 | 2.1 | 5.6 | -0.5 | 6.2 | -280.2 | 2478.0 |
| **Right Shoulder** | *tBBT* | -0.4 | 10.6 | 5.1 * | 17.9 | -1.2 | 6.6 | 4710.8 | 21875.9 |
|  | *JHFT task 2* | 0.1 | 11.2 | 1.7 | 10.0 | 2.6 | 18.5 | 15483.2 | 31764.5 |
|  | *JHFT task 4* | -0.8 | 4.7 | -3.9 | 23.0 | 1.6 | 5.6 | -1289.0 | 4972.1 |
|  | *JHFT task 7* | -4.0 | 10.5 | -4.4 | 9.7 | 0.3 | 9.3 | -887.6 | 7720.9 |
| **Torso** | *tBBT* | 2.2 | 3.9 | 6.9 * | 9.4 | 3.5 | 11.8 | 3299.1 | 9687.1 |
|  | *JHFT task 2* | 0.8 | 4.7 | 1.1 | 3.3 | 3.2 | 10.2 | 83.3 | 4851.7 |
|  | *JHFT task 4* | -2.8 * | 1.7 | -1.4 * | 3.4 | 2.9 | 9.9 | -241.6 | 682.1 |
|  | *JHFT task 7* | -0.1 | 6.2 | -1.8 | 3.7 | 2.5 | 6.8 | 9.7 | 365.3 |
