# Supplementary material for: Kinematic analysis of motor learning in upper limb body-powered bypass prosthesis training
Source: PLoS One. 2020 Jan 24;15(1):e0226563. doi: 10.1371/journal.pone.0226563 (PMC6980621; doi:10.1371/journal.pone.0226563)
Supplement: S1 File — (DOCX) [file pone.0226563.s005.docx]

**Supplemental Material**

**Functional volume**

This functional volume measure was adapted as a summary measure that could be an alternative to reporting the ranges of three DOFs in the shoulder or torso. The use of such a measure was inspired from research reporting on 2D ellipse fitting methods for Center of Pressure measurements during stance (1, 2). For those joints, an ellipsoid was fit to a 3D plot of a joint’s angular trajectory along independent axes for each DOF containing 90% of the data. The major and minor axes of the ellipsoid were used to calculate the volume and centroid coordinates. The direction of these axes or other characteristics are not provided but could be useful in determining a change in overall movement pattern and orientation. An example of a single subject’s left shoulder angular trajectory during the tBBT fitted with the ellipsoid is shown in Supplemental Figure 1.


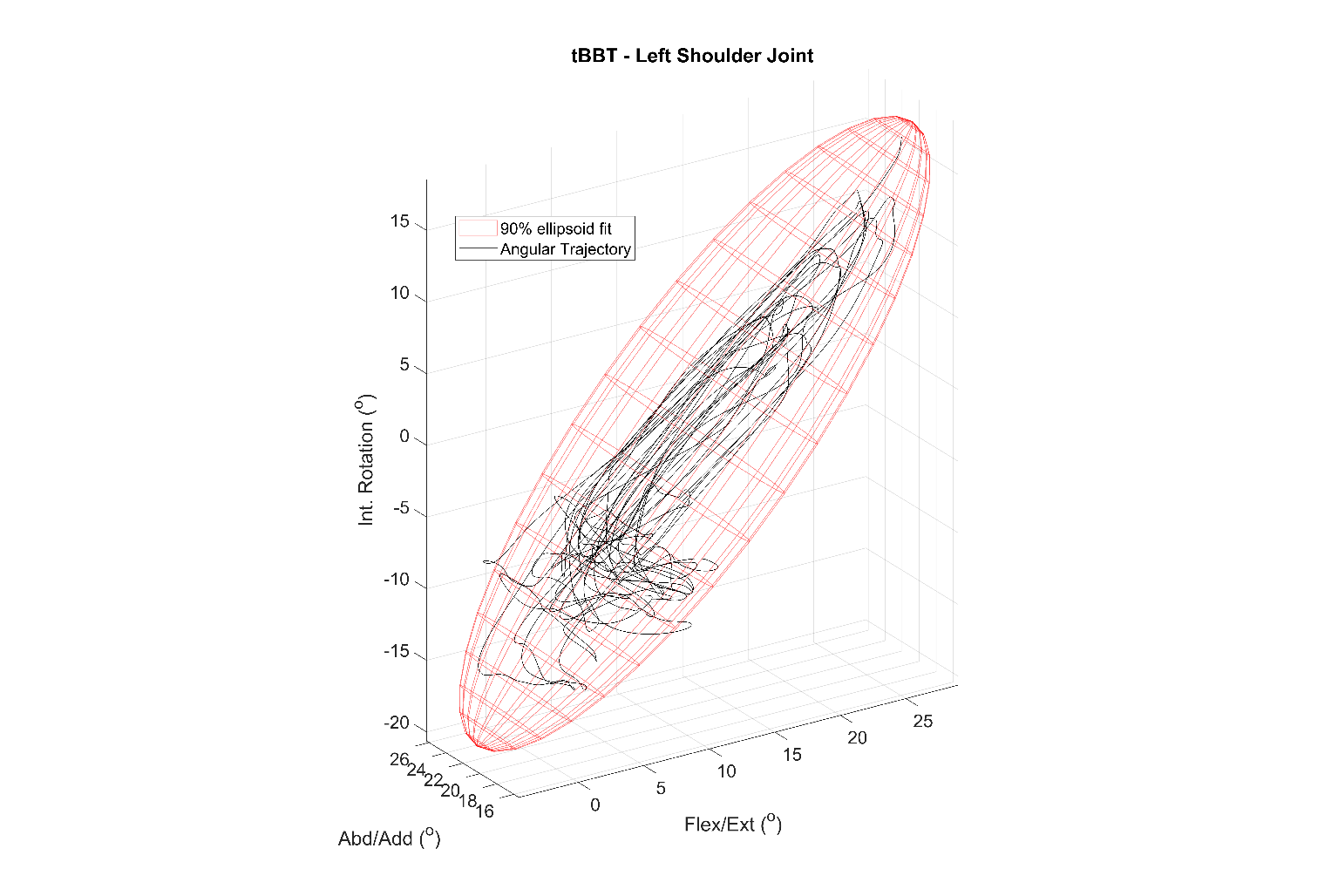


**Supplemental Figure 1:** Ellipsoid fit for a single subject’s left shoulder angular trajectory for tBBT. X, Y, and Z axes represent flexion/extension, abduction/adduction, and internal/external rotation, respectively.

The difference between the centroid location for each joint/task between session 1 and session 2 is shown in Supplemental Table 1. For each X, Y, Z coordinate, the median value of the difference and the interquartile range (IQR) is provided. The median difference in ellipsoid volume across training sessions for each joint/task is also provided. Results indicate some sensitivity to training in terms of centroid location but not in terms of volume due to high variability. As a measure, we believe this is approach is a useful summary of the joints working space but may require a larger sample to validate and establish.

**Supplemental Table 1:** Median difference of ellipsoid centroid location and volume for each joint and task. Significance between motion capture Session 1 and Session 2 is indicated by a * signifying a p-value < 0.05.

|  |  | **Difference in centroid** | | | | | | **Difference in volume** | |
| --- | --- | --- | --- | --- | --- | --- | --- | --- | --- |
|  |  | *x med* | *x IQR* | *y med* | *y IQR* | *z med* | *z IQR* | *med* | *IQR* |
| **Left Shoulder** | *tBBT* | 8.3 * | 9.5 | 2.6 * | 3.8 | -7.0 | 8.8 | -672.7 | 10373.3 |
|  | *JHFT task 2* | 12.6 | 13.4 | 4.0 | 9.4 | 4.8 | 30.1 | 4209.3 | 9929.7 |
|  | *JHFT task 4* | 3.5 | 10.1 | -2.5 | 11.5 | 5.5 | 25.8 | -53.7 | 750.5 |
|  | *JHFT task 7* | 3.1 * | 6.3 | 2.1 | 5.6 | -0.5 | 6.2 | -280.2 | 2478.0 |
| **Right Shoulder** | *tBBT* | -0.4 | 10.6 | 5.1 * | 17.9 | -1.2 | 6.6 | 4710.8 | 21875.9 |
|  | *JHFT task 2* | 0.1 | 11.2 | 1.7 | 10.0 | 2.6 | 18.5 | 15483.2 | 31764.5 |
|  | *JHFT task 4* | -0.8 | 4.7 | -3.9 | 23.0 | 1.6 | 5.6 | -1289.0 | 4972.1 |
|  | *JHFT task 7* | -4.0 | 10.5 | -4.4 | 9.7 | 0.3 | 9.3 | -887.6 | 7720.9 |
| **Torso** | *tBBT* | 2.2 | 3.9 | 6.9 * | 9.4 | 3.5 | 11.8 | 3299.1 | 9687.1 |
|  | *JHFT task 2* | 0.8 | 4.7 | 1.1 | 3.3 | 3.2 | 10.2 | 83.3 | 4851.7 |
|  | *JHFT task 4* | -2.8 * | 1.7 | -1.4 * | 3.4 | 2.9 | 9.9 | -241.6 | 682.1 |
|  | *JHFT task 7* | -0.1 | 6.2 | -1.8 | 3.7 | 2.5 | 6.8 | 9.7 | 365.3 |

**Pearson’s correlation coefficient**

Pearson’s correlation coefficient has been used to analyze similar joint angle trajectories in gait studies as a comparison of temporal aspects of movement. Here in the upper limb, a linear fit method correlated the 1^st^ derivative of each DOF angular displacement for Session 1 and Session 2. Individual DOFs were required in this analysis due to the importance of direction in this temporal analysis. This method assesses waveform shape similarity between sessions, independent of amplitude or mean difference, therefore quantifying how similar the timing and direction of joint movements are between motion capture sessions (3-5).

Results of our correlation analysis provide a unique summary of the temporal differences in joint movements that may capture user’s changing strategies. From results presented here in Supplemental Figure 2, it is clear some changes in movement can be attributed to conscious changes in approach and some are due to unconscious changes in motor control. A temporal summary, such as this measure, may be useful in differentiating conscious changes (uncorrelated) in other measures and others (correlated).

**
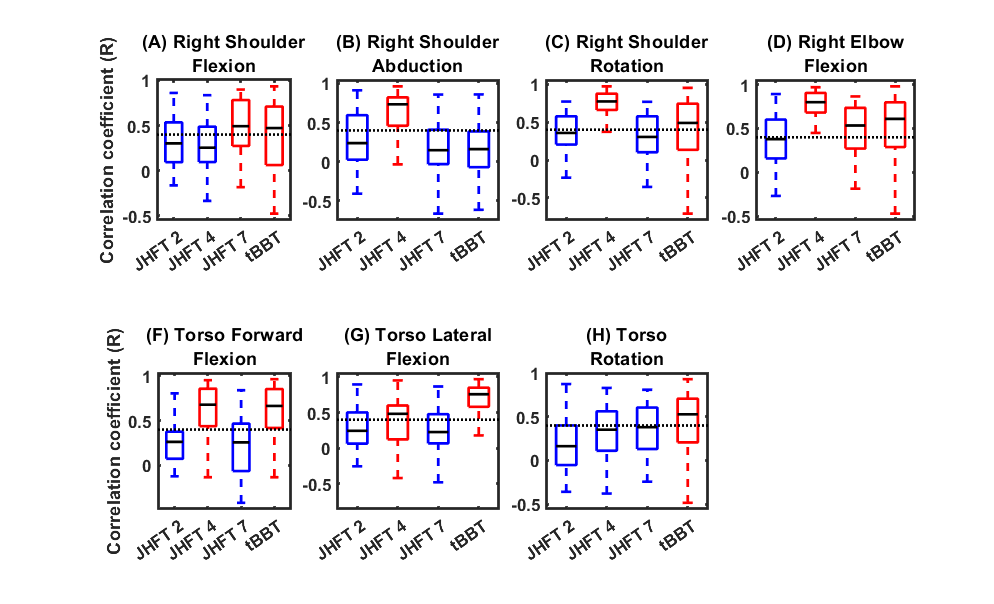
**

**Supplmental Figure 2: Pearson’s Correlation Coefficient*.*** Linear fit comparisons between sessions to generate R values summarized as a boxplot. The dotted line in each plot at y = 0.4 represents the R-value threshold above which waveforms are considered moderately to strongly correlated. Blue boxes indicate medians below threshold, or dissimilar strategies, red boxes indicate medians above threshold, or similar strategies. X axis ticks indicate JHFT task number (2, 4, and 7) followed by tBBT.

**Path integral**

Path integral is a novel metric designed to summarize both the displacement from the neutral joint angle as well as the time spent at that angle. Specifically, the calculation determines the area under the resultant angular trajectory for a segment using a trapezoidal approximation. Results shown in supplemental figure 3 are normalized before the analysis by matching number of samples between sessions within joints and tasks.

This measure could be useful as a time-weighted summary of the angular position of a joint, similar to how maximum angle is used to measure compensation. We believe this additional information provides a better summary of not just the angles used but how common that angle was while performing a specific task.

**
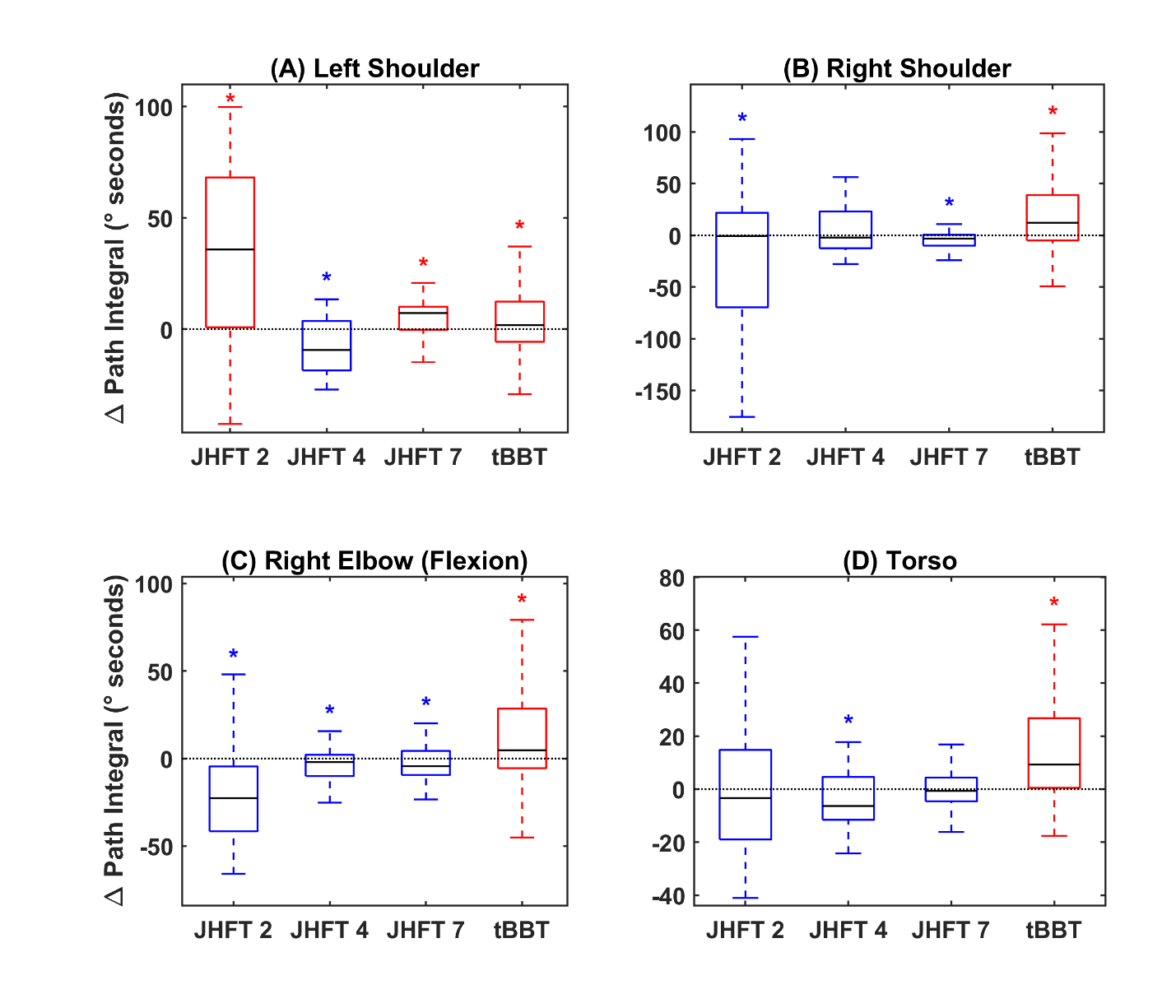
**

**Supplemental Figure 3: Path integral.** Difference in path integral between sessions summarized as a boxplot. Blue boxes indicate medians below zero, or reduced effort, red boxes indicate medians above zero, or increased effort. X axis ticks indicate JHFT task number (2, 4, and 7) followed by tBBT.

Supplemental References

1. Lee G, Park AE. Development of a more robust tool for postural stability analysis of laparoscopic surgeons. Surg Endosc. 2008;22(4):1087-92.

2. Kontson K, Marcus I, Myklebust B, Civillico E. An integrated movement analysis framework to study upper limb function: A pilot study. IEEE Transactions on Neural Systems and Rehabilitation Engineering. 2017;25(10):1874-83.

3. Picerno P, Cereatti A, Cappozzo A. Joint kinematics estimate using wearable inertial and magnetic sensing modules. Gait Posture. 2008;28(4):588-95.

4. Kadaba MP, Ramakrishnan HK, Wootten ME, Gainey J, Gorton G, Cochran GV. Repeatability of kinematic, kinetic, and electromyographic data in normal adult gait. J Orthop Res. 1989;7(6):849-60.

5. Lighthall Haubert L, Mulroy SJ, Requejo PS, Maneekobkunwong S, Gronley JK, Rankin JW, et al. Effect of reverse manual wheelchair propulsion on shoulder kinematics, kinetics and muscular activity in persons with paraplegia. The journal of spinal cord medicine. 2019:1-13.
